# Predictors of Intensive Care Unit Admission among Hospitalized COVID-19 Patients in a Large University Hospital in Tehran, Iran

**DOI:** 10.34172/jrhs.2021.44

**Published:** 2021-02-21

**Authors:** Hossein Hatami, Hussein Soleimantabar, Mehrdad Ghasemian, Negar Delbari, Shayan Aryannezhad

**Affiliations:** ^1^Department of Public Health, School of Public Health and Safety and Environmental and Occupational Hazards Control Research Center, Shahid Beheshti University of Medical Sciences, Tehran, Iran; ^2^Department of Radiology, School of Medicine, Imam Hossein Hospital, Shahid Beheshti University of Medical Sciences, Tehran, Iran; ^3^Imam Hossein Hospital, Shahid Beheshti University of Medical Sciences, Tehran, Iran; ^4^School of Public Health and Safety, Shahid Beheshti University of Medical Sciences, Tehran, Iran

**Keywords:** SARS-CoV-2, COVID-19, Intensive Care Units, Risk factors, Critical care

## Abstract

**Background:** The rapid increase in the spread of COVID-19 and the numbers of infected patients worldwide has highlighted the need for intensive care unit (ICU) beds and more advanced therapy. This need is more urgent in resource-constrained settings. The present study aimed to identify the predictors of ICU admission among hospitalized COVID-19 patients.

**Study design:** The current study was conducted based on a retrospective cohort design.

**Methods:** The participants included 665 definite cases of severe acute respiratory syndrome coronavirus 2 (SARS-CoV-2) hospitalized in Imam Hossein Hospital from February 20 to May 14, 2020. The baseline characteristics of patients were assessed, and multivariate logistic regression analysis was utilized to determine the significant odds ratio (OR) for ICU admission.

**Results:** Participants were aged 59.52±16.72 years, and the majority (55.6%) of them were male. Compared to non-ICU patients (n=547), the ICU patients (n=118) were older, had more baseline comorbidities, and presented more often with dyspnea, convulsion, loss of consciousness, tachycardia, tachypnea, and hypoxia, and less often with myalgia. Significant OR (95% CI) of ICU admission was observed for the 60-80 age group (2.42, 95%CI: 1.01; 5.79), ≥80 age group (3.73, 95%CI: 1.44; 9.42), ≥3 comorbidities (2.07, 95%CI: 1.31; 3.80), loss of consciousness (6.70, 95%CI: 2.94, 15.24), tachypnea (1.79, 95%CI: 1.03, 3.11), and SpO2<90 (5.83, 95%CI: 2.74; 12.4). Abnormal laboratory results were more common among ICU-admitted patients; in this regard, leukocytosis (4.45, 95%CI: 1.49, 13.31), lymphopenia (2.39, 95%CI: 1.30; 4.39), elevated creatine phosphokinase (CPK) (1.99, 95%CI: 1.04; 3.83), and increased aspartate aminotransferase (AST) (2.25, 95%CI: 1.18-4.30) had a significant OR of ICU admission. Chest computer tomography (CT) revealed that consolidation (1.82, 95%CI: 1.02, 3.24), pleural effusion (3.19, 95%CI: 1.71, 5.95), and crazy paving pattern (8.36, 95%CI: 1.92, 36.48) had a significant OR of ICU admission.

**Conclusion:** As evidenced by the obtained results, the predictors of ICU admission were identified among epidemiological characteristics, presenting symptoms and signs, laboratory tests, and chest CT findings.

## Introduction

The coronavirus disease 2019 (COVID-19) caused by severe acute respiratory syndrome coronavirus 2 (SARS-CoV-2) was first reported in Wuhan, China, in December 2019. It rapidly spread all over the world and was declared as a pandemic posing a major threat to public health ^1^. By the end of January 2021, more than 101.5 million infected cases and 2.1 million deaths have been attributed to this disease across the globe ^2^.

The rate of intensive care unit (ICU) admission among hospitalized infected patients has been reported to be up to 30% ^3^. The rapid increase in disease spread and the numbers of infected patients worldwide has highlighted the need for ICUs and more advanced therapy. This need is more urgent in the resource-constrained settings which suffer from shortage in ICU and ventilator capacity ^4,5^. Therefore, the identification of risk factors associated with disease severity may help better resource allocation. Moreover, due to rapid disease progression ^6^, an awareness of the risk factors associated with poor outcomes would also help clinicians in early identification and better timely intervention of patients who would require advanced care during hospitalization. The risk factors associated with poor outcomes may also be used for the development of risk stratification models applicable in practice and also new standard thresholds for ICU admissions^7,8^.

Several clinical features have shown an association with ICU admission and severe outcomes among infected patients^9,10^. A systematic review study indicated that male gender among demographic data, dyspnea among signs and symptoms, as well as chronic obstructive pulmonary disease (COPD), cardiovascular disease (CVD), and hypertension (HTN) among comorbidities, were strongly associated with ICU admission ^9^. Other clinical and laboratory predictive factors for ICU admission include older age, tachypnea, low pulse oxygen saturation, smoking history, low lymphocyte count, high lactate dehydrogenase, and procalcitonin levels greater than C-reactive protein (CRP) values ^7,11^. Moreover, radiological examination, especially chest computer tomography (CT) plays a key role in the early detection of COVID-19. Furthermore, chest CT findings have prognostic utility for predicting the progression risk of patients at the time of admission and further need for ICU admission ^12,13^.

Risk factors for ICU admission have not yet been validated in different populations, especially in low-income nations. These communities may be distinctive in terms of lifestyle, health-seeking behaviors, accessibility to high-quality health services, accurate symptom description, comorbid conditions, as well as ICU bed and ventilator capacity, which would affect ICU admission decision-making. Therefore, further investigation is required, particularly in Iran which is currently experiencing one of the highest mortality rates in the world ^14^. The current study aimed to identify the key predictors of ICU admission among hospitalized COVID-19 patients in a large university hospital in Tehran, Iran.

## Methods

###  Study participants

A total of 2643 suspected SARS-CoV-2 cases were admitted to Imam Hossein Hospital, a large university hospital located in Tehran, Iran, during the first peak of the COVID-19 surge in Iran from February 20 to May 14, 2020 ^15^. Hospital admission was based on the clinical judgment of an emergency physician. Definite hospitalized COVID-19 cases (based on positive reverse transcription-polymerase chain reaction [RT-PCR] assays) (n=691) were first enrolled. Finally, 665 cases were entered into this retrospective observational analytic study after the exclusion of cases aged<18 (n=7) and those with missing data (n=19).

###  Data collection

 At the emergency department of the hospital, demographic data, as well as history and physical examination, were recorded, and blood samples were obtained and sent for laboratory tests. Nasopharyngeal swabs specimens were taken from suspected patients, followed by reverse transcription-polymerase chain reaction (RT-PCR). Upon admission, the patients underwent a low-dose chest CT scan in the supine position and at full inspiration without contrast medium injection. Chest CT scans were performed using a 16 detector CT scanner. Experienced radiologists evaluated and recorded imaging features, including the pattern of alternations (i.e. ground glass opacification [GGO], consolidation, nodule), distribution (peripheral/central, unilateral/bilateral, multifocal/unifocal), and associated findings (pleural and pericardial effusion, tree-in-bud and crazy paving pattern).

###  Definitions

Confirmed infection was defined as a person with at least one positive nasopharyngeal SARS-CoV-2 RT-PCR test result^16^. The participants were assigned to two groups based on ICU admission during their hospitalization. Neutrophil count and lymphocyte count were calculated by multiplying the percentages of neutrophils and lymphocytes by the total white blood cell (WBC) count, respectively. Regarding radiologic characteristics, ground-glass opacity (GGO) was considered a hazy increased lung opacity area which does not obscure the underlying bronchial structures and vessels. Moreover, the location of the lesion was considered central if the lesion location was limited to the bronchi, trachea, or segmental bronchi; otherwise, it was considered peripheral^17^. The “tree-in-bud” pattern refers to a small soft-tissue centrilobular nodule linked to multiple branching linear structures of similar caliber originating from a single stalk ^18^. “Crazy paving” was also considered the appearance of scattered GGO with superimposed interlobular and interlobular septal thickening ^19^.

###  Statistical analysis

 Data were expressed as mean ±standard deviation, median, and interquartile range (IQR) for quantitative variables and percentages for categorical variables. The comparison between ICU and non-ICU groups was made by independent t-test and Mann-Whitney U test for normally and non-normally distributed quantitative variables, respectively. Moreover, it was carried out by chi-square or two-tailed Fisher's exact test for categorical variables. A p-value less than 0.05 was considered statistically significant. The variables were categorized into four groups (namely demographic, clinical, laboratory, and radiologic). In each group, categorical variables with a significant difference between ICU and non-ICU were entered in univariate logistic regression analysis. Variables with significant between-group differences in univariate models entered in multivariate logistic regression analysis for the identification of demographic, clinical, laboratory, and radiologic risk factors associated with ICU admission. All the statistical analyses were performed in SPSS software (version 19.0).

## Results

The present study was conducted on 665 subjects aged 59.52±16.72. In terms of gender, 44.4% of cases were female. They either ended up in admission to the general ward (n=547) or the ICU (n=118) (Table 1). Compared to patients admitted to the general ward, ICU-admitted patients were older (66.82 ±14.82 vs. 57.94 ±16.70). Most prevalent baseline comorbidities were hypertension (33.5%), diabetes mellitus (27.5%), and cardiovascular disease (CVD) (19.6%). The prevalence rates of underlying lung diseases were obtained at 3.9% and 2.0% for asthma and COPD. The ICU-admitted patients had a higher prevalence of the mean number of baseline comorbidities, compared to others (1.55±1.12 vs. 1.23±1.25). Further multivariate logistic regression analysis of variables revealed significant odds ratios for the need for ICU admission in those who were in the 60-80 age group (2.42, 95%CI: 1.01, 5.79), older than 80 (3.73, 95%CI: 1.44, 9.42), and those with three or more baseline comorbidities (2.07, 95%CI: 1.31, 3.80).

**Table 1:** Demographic and epidemiologic characteristics of hospitalized COVID-19 patients

**Variables**	**All patients (n=665)**		**General ward (n=547)**		**ICU (n=118)**		
	**Number**	**Percent**	**Number**	**Percent**	**Number**	**Percent**	***P*** **-value**
Sex							0.560
Male	370	55.6	295	53.9	75	63.6	
Female	295	44.4	252	46.1	43	36.4	
Age (yr)							0.001
<40	89	13.4	82	15.0	7	5.9	
40-60	230	34.6	203	37.1	27	22.9	
60-80	257	38.6	200	36.6	57	48.3	
≥80	89	13.4	62	11.3	27	22.9	
Body mass index (kg/m^2^)							0.198
Non-obese (<30)	236	68.0	186	70.5	50	78.5	
Obesity (≥30)	92	28.0	78	29.5	14	21.5	
Co-morbidities							
Diabetes mellitus							0.116
No	481	72.5	403	83.8	78	66.7	
Yes	182	27.5	143	26.2	39	33.3	
Hypertension							0.001
No	440	66.5	378	69.2	62	53.4	
Yes	222	33.5	168	30.8	54	46.6	
Cardiovascular disease							0.018
No	532	80.4	448	82.1	84	72.4	
Yes	130	19.6	98	17.9	32	27.6	
Chronic kidney disease							0.816
No	614	92.7	507	92.9	107	92.2	
Yes	48	7.3	39	7.1	9	7.8	
Chronic obstructive pulmonary disease							0.259
No	649	98.0	537	98.4	112	96.6	
Yes	13	2.0	9	1.6	4	3.4	
Asthma							0.193
No	636	96.1	527	96.5	109	94.0	
Yes	26	3.9	19	3.5	7	6.0	
Malignancy							0.628
No	609	92.0	501	91.8	108	93.1	
Yes	53	8.0	45	8.2	8	6.9	
Immunosuppression							0.161
No	606	91.5	496	90.8	110	94.8	
Yes	56	8.5	50	9.2	6	5.2	
Cerebrovascular accident							0.029
No	632	95.3	525	96.2	107	91.5	
Yes	31	4.7	21	3.8	10	8.5	
Pregnancy							0.223
No	652	98.5	536	98.2	116	100.0	
Yes	10	1.5	10	1.8	0	0.0	
Numbers of co-morbidities							0.001
0	222	33.4	201	36.7	21	17.8	
1	184	27.7	143	26.1	41	34.7	
2	150	22.6	119	21.8	31	26.3	
≥3	109	16.4	84	15.4	25	21.2	

The most prevalent presenting symptoms were reported as cough (62.6%), fever (55.9%), and dyspnea (52.6%) in all patients (Table 2). Dyspnea, convulsion, and loss of consciousness were more common in the ICU-admitted patients, whereas myalgia was more common in patients admitted to the general hospital ward. Regarding baseline vital signs, pulse rate (93.91 ±18.90 vs. 87.94 ±14.00) and respiratory rate (21.12 ±5.61 vs. 18.81 ±3.97) were higher in the ICU-admitted patients, compared to those reported in non-ICU patients. Nonetheless, ICU-admitted patients had lower SpO2 (84.35 ±10.96 vs. 91.11 ±5.60) and were more frequently presented with tachycardia, tachypnea, and hypoxia (SpO2 <90 and 90≤ SpO2 <95). Significant odds ratios for the need for ICU admission were observed for loss of consciousness (6.70, 95%CI: 2.94, 15.24), tachypnea (1.79, 95%CI: 1.03, 3.11), and SpO2 <90 (5.83, 95%CI: 2.74, 12.4). On the other hand, patients with myalgia as a presenting symptom had lower odds of ICU admission (0.52, 95%CI: 0.31, 0.87).

Hematologic tests upon admission showed leukocytosis (16.3%), neutrophilia (20.9%), lymphopenia (34.5%), and anemia (32.2%) which were all higher in the ICU group, compared to the non-ICU group (P< 0.05). In terms of infection-related parameters, 85.5% of patients had increased CRP (higher in the ICU group), and 80.1% of cases had increased erythrocyte sedimentation rate (ESR). Coagulatory tests pointed to increased Prothrombin time (PT) (21.4%) and international normalized ratio (INR) (20.1%) which were more frequently observed in the ICU group. Venous blood gas (VBG) results confirmed lower pH in the ICU group, compared to the non-ICU patients. Other laboratory tests demonstrated a higher prevalence of abnormal results (increased urea, creatinine, sodium, Creatine phosphokinase [CPK], Aspartate aminotransferase [AST], alanine aminotransferase [ALT], Lactate dehydrogenase [LDH], and decreased sodium) in the ICU group, compared to the non-ICU patients (Table 3). Four abnormal tests at baseline were associated with significance odds of ICU admission: leukocytocis (4.45, 95%CI: 1.49, 13.31), lymphopenia (2.39, 95%CI: 1.30, 4.39), increased CPK (1.99, 95%CI: 1.04-3.83), and increased AST (2.25, 95%CI: 1.18, 4.30).

**Table 2:** Clinical characteristics of hospitalized COVID-19 patients

**Variables**	**All patients (n=665)**		**General ward (n=547)**		**ICU (n=118)**		***P*** **-value**
	**Number**	**Percent**	**Number**	**Percent**	**Number**	**Percent**	
**Symptoms**							
Cough							0.236
No	248	37.4	199	36.4	49	19.8	
Yes	415	62.6	348	63.6	67	57.8	
Fever							0.391
No	292	44.1	245	44.9	47	40.5	
Yes	370	55.9	301	55.1	69	59.5	
Dyspnea							0.001
No	315	47.4	277	50.6	38	32.5	
Yes	349	52.6	270	49.4	79	67.5	
Myalgia							0.012
No	376	56.7	298	54.5	78	67.2	
Yes	287	43.3	249	45.5	38	32.8	
Fatigue							0.137
No	423	63.8	342	80.9	81	69.8	
Yes	240	36.2	205	37.5	35	30.2	
Chest pain							0.727
No	547	82.5	450	82.3	97	83.6	
Yes	116	17.5	97	17.7	19	16.4	
Sweating							0.239
No	607	91.6	504	92.1	103	88.8	
Yes	56	8.4	43	7.9	13	11.2	
Anorexia							0.698
No	511	77.1	420	76.8	91	78.4	
Yes	152	22.9	127	23.2	25	21.6	
Headache							0.517
No	601	90.6	494	90.3	107	92.2	
Yes	62	9.4	53	9.7	9	7.8	
Sore throat							1.000
No	653	98.5	538	98.4	115	99.1	
Yes	10	1.5	9	1.6	1	0.9	
Diarrhea							0.391
No	623	94.0	512	93.6	11	95.7	
Yes	40	6.0	35	6.4	5	4.3	
Nausea/Vomiting							0.355
No	540	81.4	442	80.8	98	84.5	
Yes	123	18.6	105	19.2	18	15.5	
Abdominal pain							0.392
No	610	92.0	501	91.6	109	94.0	
Yes	53	8.0	46	8.4	7	6.0	
Dizziness							0.336
No	634	95.6	525	96.0	109	94.0	
Yes	29	4.4	22	4.0	7	6.0	
Convulsion							0.001
No	653	98.5	544	99.5	109	94.0	
Yes	10	1.5	3	0.5	7	6.0	
Loss of consciousness							0.001
No	615	92.6	522	95.4	93	79.5	
Yes	49	7.4	25	4.6	24	20.5	
Others							0.187
No	584	88.1	486	88.8	98	84.5	
Yes	79	11.9	61	11.2	18	15.5	
**Vital signs**							
Tachycardia							0.001
No	544	84.5	458	86.7	86	74.1	
Yes	100	15.5	70	13.3	30	25.9	
Tachypnea							0.001
No	455	79.3	386	82.3	69	65.7	
Yes	119	20.7	83	17.7	36	34.4	
Temperature (≥38.5)							0.807
No	527	83.0	434	82.8	93	83.8	
Yes	108	17.0	90	17.2	18	16.2	
O_2_ saturation (Spo2)							0.001
≥95	147	24.8	134	27.8	13	11.7	
90-94	246	41.5	219	45.4	27	24.3	
<90	200	33.7	129	26.8	71	64.0	

**Table 3:** Baseline laboratory values of hospitalized COVID-19 patients

**Variables**	**All patients** **(n=665)**		**General ward** **(n=547)**		**ICU** **(n=118)**		***P*** **-value**
	**Number**	**Percent**	**Number**	**Percent**	**Number**	**Percent**	
Leukocytes (×10^9^/L)							0.001
>11.20	105	16.3	71	13.4	34	29.1	
4.20-11.20	454	70.4	384	72.7	70	59.8	
<4.20	86	13.3	73	13.8	13	11.1	
Neutrophil count (×10^9^/L)							0.001
>7.70	134	20.9	96	18.3	38	32.5	
1.5-7.7	495	77.4	418	80.0	77	65.8	
<1.50	11	1.7	9	1.7	2	1.7	
Lymphocyte count (×10^9^/L)							0.001
>4.00	10	1.6	8	1.5	2	1.7	
1.00-4.00	404	63.9	350	67.8	54	47.0	
<1.00	221	34.5	161	30.7	60	51.3	
Platelets (×10^9^/L)							0.253
>450.0	176	27.3	137	25.9	39	33.3	
150.0-450.0	454	70.4	379	71.8	75	64.1	
<150.0	15	2.3	12	2.3	3	2.6	
Hemoglobin (g/dL)							0.475
≥12	437	67.8	361	68.4	76	65.0	
<12	208	32.2	167	31.6	41	35.0	
CRP (mg/L)							0.268
>10	511	85.0	412	84.3	99	88.4	
≤10	90	15.0	77	15.7	13	11.6	
ESR (mm/h)							0.975
>20	330	80.1	253	80.1	77	80.2	
≤20	82	19.9	63	19.9	19	19.8	
INR							0.027
>1.26	82	20.1	54	17.6	28	27.7	
≤1.26	326	79.9	253	82.4	73	72.3	
Prothrombin time (s)							0.032
>13.6	87	21.4	58	18.9	29	29.0	
≤13.6	320	78.6	249	81.1	71	71.1	
Partial thrombin time (s)							0.596
>40	20	5.0	14	4.6	6	5.9	
≤40	384	95.5	289	95.4	05	94.1	
PH							0.001
>7.41	328	53.9	269	54.7	59	50.4	
7.31-7.41	242	29.7	202	33.2	40	34.1	
<7.31	39	6.4	21	4.3	18	15.5	
PCO_2_ (mmHg)							0.633
>52.0	76	12.5	60	12.2	16	13.7	
40.0-52.0	326	53.5	268	54.5	58	49.6	
<40.0	207	34.0	164	33.3	43	36.8	
HCO_3_ (mEq/L)							0.254
>27.00	306	50.2	251	51.0	55	47.0	
22.0-27.0	231	37.9	188	38.2	43	36.8	
<22.00	72	11.8	53	10.8	19	16.2	
Urea (mg/dl)							0.001
>45.0	202	31.7	149	28.7	53	45.3	
≤45.0	435	68.3	371	85.3	64	54.7	
Serum creatinine (mg/dl)							0.001
>1.5	128	20.1	92	17.7	36	31.0	
≤1.5	509	79.9	429	82.3	80	69.0	
Serum sodium (mmol/L)							0.001
>146.00	5	0.8	1	0.2	4	3.4	
133.0-146.0	528	86.0	438	72.8	90	77.6	
<133.00	81	13.2	59	11.8	22	19.0	
Serum potassium (mmol/L)							0.482
>5.00	49	8.0	38	7.6	11	9.5	
3.80-5.00	446	72.8	359	72.2	87	75.0	
<3.80	118	19.2	100	20.1	18	15.5	
CPK (IU/L)							0.001
>195.0	173	32.1	124	28.4	49	48.0	
≤195.0	366	67.9	313	71.6	53	52.0	
AST (U/L)							0.001
>40.0	137	36.6	92	31.6	45	54.2	
≤40.0	238	63.4	200	68.4	38	45.8	
ALT (U/L)							0.050
>50.0	60	16.1	41	14.1	19	23.2	
≤50.0	312	83.9	249	85.9	63	76.8	
LDH (U/L)							0.031
>460.0	168	66.7	119	63.0	49	77.8	
≤460.0	84	33.3	70	37.0	12	22.2	

**Figure 1:**
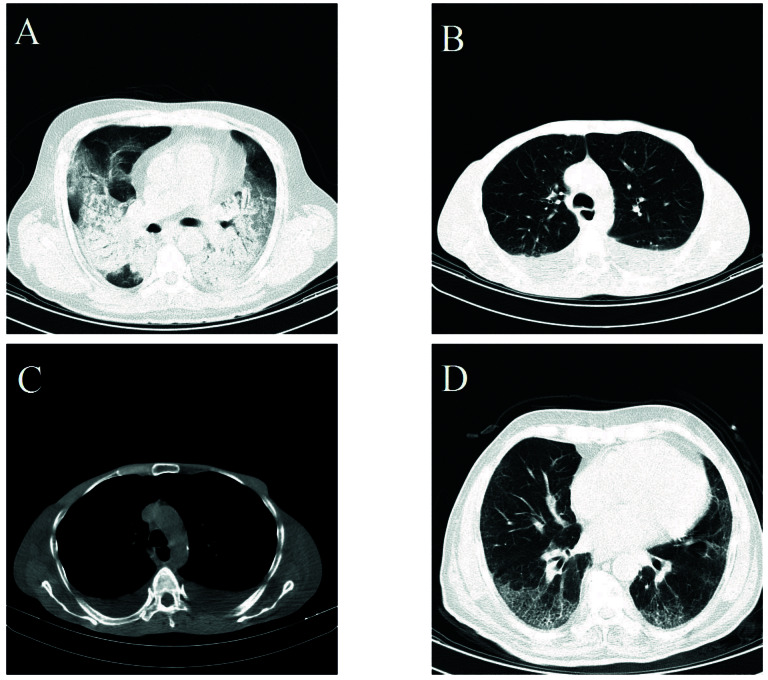
Spiral chest CT scan of SARS-CoV2 infected patients. A 40- year-old woman with bilateral GGO and consolidation (A). A 56-year-old man with bilateral pleural effusion apparent at lung view (B) and mediastinal view (C). A 75-year-old man with bilateral ground glass opacity combined with interlobular septal thickening making a Crazy Paving appearance (D).

Normal CT scan findings were observed only in the non-ICU patients (6.2%; Table 4). The most prevalent radiologic lesion was GGO (observed in 80.3% of patients), and the distribution of lesions were mainly bilateral (84.2%), peripheral (74.3%), and multifocal (72.5%). Findings which were more frequently observed in the ICU patients included consolidation, pleural effusion, cardiomegaly, aortocoronary calcification, and crazy paving pattern. The most prevalent combination of findings were GOO + consolidation (10.4%), GOO + pleural effusion (9.0%), and consolidation + pleural effusion (5.0%) respectively. The results of multivariate logistic regression analysis showed significant odds of ICU admission for consolidation (1.82, 95%CI: 1.02, 3.24), pleural effusion (3.19, 95%CI: 1.71, 5.95), and crazy paving pattern (8.36, 95%CI: 1.92, 36.48) (Figure 1).

The overall mortality rate was 19.1% (76.3% in the ICU group and 6.3% in the non-ICU group; *P*<0.001). Compared to general ward patients, ICU-admitted patients had a longer duration of hospitalization (9.73 ±7.49 vs. 5.27 ±4.71; *P*<0.001), a higher rate of invasive mechanical ventilation (IMV) (67.8% vs. 3.7%; *P*<0.001), non-invasive mechanical ventilation (NIMV) (21.4% vs. 2.6%, *P*<0.001), dialysis, tracheostomy, and other procedures (Supplementary Table 1).

## Discussion

 To the best of our knowledge, this is the first study to assess the predictors of ICU admission in a sample of hospitalized COVID-19 patients in a developing country. In the present study, 17.7% of patients were admitted to ICU, and they faced a high mortality rate (76.3%), indicating the severity and highly progressed state of their disease course. Multivariate logistic regression analysis revealed the best predictors of ICU admission among epidemiological characteristics (age of over 80 years, age of over 60 years, and having more than three baseline co-morbidities). The best predictors of ICU admission among presenting signs and symptoms are loss of consciousness, SpO2 <90, and tachypnea. Leukocytosis, lymphopenia, increased AST, and elevated creatine phosphokinase (CPK), as well as radiologic findings of chest CT scan (including crazy paving pattern, pleural effusion, and consolidation), can also predict ICU admission.

Medical history of diabetes mellitus (DM), HTN, CVD, cerebrovascular accident (CVA), and COPD have been cited as predictive factors for severe outcomes in COVID-19 patients ^9,20^. The present study also found significantly higher rates of HTN, CVD, and, CVA in ICU rather than non-ICU patients. Nevertheless, none of the underlying diseases had an independent significant association with ICU admission in multivariate analysis in the current study. However, having multiple comorbidities was found to predict ICU admission (more than three underlying comorbidities were two times more associated with ICU admission, irrespective of the underlying disease nature). The findings of the current study are in line with those reported by Carlino et al. who showed that none of the underlying medical conditions alone could predict ICU admission with a good accuracy ^21^.

The well-established association of age with severe outcomes, such as ICU admission in COVID-19 infected patients, had been attributed to the increased number of comorbidities in older people ^7,11^. However, the present study suggested that patients aged 60-80 years (OR= 2.42, 95%CI 1.01-5.79) and over 80 years (OR=3.73, 95%CI 1.44-9.42) are at higher risk of disease deterioration and ICU admission independently from other cofounders, such as underlying comorbidities. It has been proposed that underlying mechanisms associated with impaired cell-mediated and humoral immune systems, as well as dysfunctional pro-inflammatory responses, might play a role in consequent severe outcomes, such as ICU admission in older adults ^22^.

**Table 4:** Radiologic findings of hospitalized COVID-19 patients

**Variables**	**All patients (n=480)**		**General ward (n=390)**		**ICU (n=90)**		***P*** **-value**
	**Number**	**Percent**	**Number**	**Percent**	**Number**	**Percent**	
Normal CT finding							0.013
No	456	95.0	366	93.8	90	100.0	
Yes	24	5.0	24	6.2	0	0.0	
Ground glass opacification (GGO)							0.702
No	94	19.7	75	19.3	19	21.1	
Yes	384	80.3	313	80.7	71	78.9	
Consolidation							0.001
No	392	81.8	330	84.4	62	68.9	
Yes	87	18.2	59	15.2	28	31.1	
Nodules							0.800
No	452	94.6	366	94.3	86	95.6	
Yes	26	5.4	22	5.7	4	4.4	
Bilateral lesion distribution							0.179
No	75	15.8	65	16.8	10	11.1	
Yes	401	84.2	321	83.2	80	88.9	
Peripheral lesion distribution							0.965
No	122	25.7	99	25.8	23	25.6	
Yes	352	74.3	285	74.2	67	74.4	
Multifocal lesion distribution							0.740
No	130	27.5	104	27.2	26	28.9	
Yes	343	72.5	279	72.8	64	71.1	
Pleural effusion							<0.001
No	418	87.4	354	91.2	64	71.1	
Yes	60	12.6	34	8.8	26	28.9	
Pericardial effusion							0.238
No	473	99.0	385	99.2	88	97.8	
Yes	5	1.0	3	0.8	2	2.2	
Cardiomegaly							0.001
No	413	86.4	345	88.9	68	75.6	
Yes	65	13.6	43	11.1	22	24.4	
Lymphadenopathy							0.839
No	425	89.3	345	89.1	80	89.9	
Yes	51	10.7	42	10.9	9	10.1	
Aortocoronary calcification							0.018
No	369	77.7	308	79.8	61	68.2	
Yes	106	22.3	78	20.2	28	31.8	
Tree in bud							1.000
No	461	96.6	374	96.6	87	96.7	
Yes	16	3.4	13	3.4	3	3.3	
Crazy paving							0.002
No	467	98.1	384	99.2	83	93.3	
Yes	9	1.9	3	0.8	6	6.7	
GGO + Consolidation							0.077
No	430	89.6	355	90.8	75	84.4	
Yes	50	10.4	36	9.2	14	15.6	
GGO + Pleural effusion							0.001
No	435	91.0	363	93.8	72	78.9	
Yes	43	9.0	24	6.2	19	21.1	
Consolidation + Pleural effusion							0.001
No	456	95.0	381	96.7	75	87.8	
Yes	24	5.0	13	3.3	11	12.2	
Bilateral GGO							0.946
No	116	24.2	94	24.1	22	24.4	
Yes	364	75.8	296	75.9	68	75.6	
Bilateral Consolidation							0.005
No	408	85.0	341	87.2	67	75.4	
Yes	72	15.0	50	12.8	22	24.6	
Bilateral Nodules							0.748
No	469	96.9	381	96.7	88	97.8	
Yes	15	3.1	13	3.3	2	2.2	

In accordance with current knowledge, those with higher respiratory rates at presentation were at increased risk for severe outcomes ^11,21^. Moreover, in line with the previous literature, SpO2<90% was the most strong predictive vital sign for ICU admission (OR=5.83, 95% CI: 2.74; 12.4) ^7,23^. This finding confirms the notion that lung involvement in the form of pneumonia (interstitium inflammation and alteration of the alveolar ventilation) and consequent hypoxemia is the main pathophysiological mechanism of the disease in critical patients ^24^ and is present in the ICU group since their admission to the emergency department.

Dyspnea as a symptom of lung involvement was significantly more common among ICU-admitted patients; however, it did not display an independent significant association with ICU admission in multivariate analysis in the current study. It could be explained by ‘silent hypoxemia’ which had been suggested in previous studies as the presence of hypoxemia without experiencing difficulty in breathing ^25,26^. Moreover, the rapid deterioration of the disease in cases of severe hypoxemia and consequent loss of consciousness was among the most significant predictors of ICU admission and severe outcomes in the present study. It can be regarded as an explanation for the observed finding since dyspnea is a purely subjective symptom ^27^. On the other hand, it was observed that myalgia is a protective factor for ICU admission (OR=0.52, 95%CI: 0.31, 0.87) probably due to the fact that patients complaining about a mild symptom, such as myalgia, are not struggling with a severe or progressive disease course.

As acknowledged in previously conducted studies, CPK level was significantly higher among the ICU group ^28^, representing an early sign of tissue injury and was associated with nearly twice-fold increased risk for ICU admission. Lactate dehydrogenase (LDH) level was also significantly higher among the ICU group; however, we failed to enter it in the multivariate analysis due to the high rate of missing data among LDH levels. Consistent with the present study, increased leukocyte count and neutrophil count were associated with ICU admission and severe outcomes in previous studies ^21^. Moreover, in the current study increased leukocyte count was among the top predictors of ICU admission (OR=4.45, 95%CI: 1.49; 13.31). Furthermore, among laboratory features, decreased lymphocyte count was also an independent predictor of ICU admission. The observed increased lymphocyte and neutrophil count, as well as decreased lymphocyte count, in the ICU group, might be an indicator of the higher systemic inflammatory response induced by the body’s cytokines ^29^. In addition, bacterial co-infections are higher among these patients and results in the aggravation of their respiratory condition. Moreover, it has been reported that lung infiltration of neutrophils and Neutrophil Extracellular Traps (NETs) observed in an autopsy specimen from a COVID-19 patient may play a role in patient deterioration and severe outcomes ^30^. Higher AST levels were also associated with a greater risk of ICU admission (OR=2.25, 95% CI: 1.18; 4.30). Although the causality between COVID-19 and liver damage is still not fully understood, the association of liver injury with severe COVID-19 infection and outcomes has been supported ^31^.

The majority of the patients had bilateral, multifocal, and peripheral lesions with a GGO pattern resembling the radiographic features related to pneumonia caused by COVID-19, and this finding was not significantly different between ICU and non-ICU patients ^32,33^. Nevertheless, it has been suggested that CT scan features upon hospital admission could predict further outcomes ^34^. In the current study, 24 patients (5.0%) had a clear chest CT, and none of them were admitted to the ICU during their hospitalization, suggesting that normal CT upon presentation is associated with a good prognosis. Moreover, three radiologic patterns (crazy-paving pattern, pleural effusion, and consolidation) were associated with ICU admission (and consequently, poor prognosis). Furthermore, the ICU-admitted patients were more likely to have a combination of CT scan findings, indicating more severe disease. Consolidation was found to be associated with severe disease and an indicator of poor outcome in the present study, as well as some previous studies ^35^. The increased rate of consolidations, along with increasing percentages of lung involvement in patients is associated with disease progression and could partially explain the observed association ^36,37^. Although crazy-paving pattern, a lesion indicative of extensive lung involvement, diffuse alveolar edema, and interstitial inflammation, is accounted as an uncommon finding even in ICU patients, it has been shown by multivariate analysis as the most strong predictors of ICU admission. However, the results of previous studies are inconsistent. Some of them pointed to an increased frequency of crazy paving patterns, along with disease progression ^37^ in ICU-admitted patients, while some others did not show any differences between different clinical groups in this regard ^38^. Pleural effusion (bilateral in 86.7% of cases), another uncommon finding, was also associated with ICU admission and poor prognosis in the present study, as well as previous studies ^13^, and it could be an indicator of bacterial co-infection. About 30% of patients with pleural effusion had underlying heart disease. The frequency of aortic calcification and cardiomegaly was found to be significantly higher in the ICU group; nonetheless, it did not display a significant OR for ICU admission.

 Regarding the notable limitations of the present study, one can refer to limited generalizability of the results since it was a retrospective study based on a single institution and it did not include patients with mild to moderate symptoms. Moreover, the obtained result might have been biased toward the overestimation of mortality, especially in the ICU-admitted group, since our hospital had a relatively high patient load and limited resources leading to restricted ICU admission criteria. Another important limitation was the high proportion of missing data in some laboratory tests (i.e. LDH which was excluded from multivariate logistic regression analysis) and the CT scan of patients. On the other hand, the strengths of the study lie in its considerable sample size and the performance of multivariate logistic regression analysis which allowed the precise identification of disease severity and ICU admission predictors.

## Conclusion

 The risk factors for critically ill COVID-19 patients requiring ICU admission must be identified to allow better recognition of the most vulnerable target group of the disease, especially in the developing countries facing limited ICU beds and more complex resource allocation problems. The present study determined the best epidemiologic, as well as clinical and paraclinical predictors of ICU admission of hospitalized COVID-19 patients, facilitating decision making of frontline physicians to stratify high-risk patients in need of intensive care.

## Acknowledgements

 The authors' deepest appreciation goes to the staff and patients of Imam Hossein Hospital who took part in this research project. Our sincere gratitude is also extended to Professor Amir Kavousi for his guidance in statistical analysis and Mrs. Soheila Rahavard who participated in the data entry process. This study was extracted from an MPH dissertation submitted by Shayan Aryannezhad to the School of Public Health and Safety, Shahid Beheshti University of Medical Sciences (IR.SBMU.PNHS.REC.1399.028).

## Conflict of interest

 The authors declare that they have no conflict of interest regarding the publication of the current article.

## Funding

 The research team did not receive any grant from organizations in the public or private sectors.

## Highlights

- Developing countries challenged by COVID-19 are facing limited intensive care unit (ICU) beds and resources.
- Older age and having more than three comorbidities predict ICU admission.
- Loss of consciousness, SpO2 <90, and tachypnea predict ICU admission.
- Leukocytosis, lymphopenia, increased aspartate aminotransferase, and elevated creatine phosphokinase can predict ICU admission.
- Crazy paving pattern, pleural effusion, and consolidation predict ICU admission.
